# Fabrication of Macroporous Biomorphic SiC from Cellulose Nanofibers Aerogel

**DOI:** 10.3390/ma11122430

**Published:** 2018-11-30

**Authors:** Mingjie Wang, Fu Liu, Yao Chen, Jianmin Gao

**Affiliations:** MOE Key Laboratory of Wooden Materials Science and Application, College of Materials Science and Technology, Beijing Forestry University, Beijing 100083, China; mannixwang@bjfu.edu.cn (M.W.); liufu2015@bjfu.edu.cn (F.L.)

**Keywords:** biomorphic, ceramics, porous materials, wood, SiC

## Abstract

The biomorphic Silicon Carbide (BioSiC) ceramic with highly interconnected porous three-dimensional (3D) structure was fabricated by utilizing balsa wood cellulose nanofibers aerogel as the biotemplate and polycarbosilane (PCS) as the preceramic precursor. Evolution of morphology, composition, and pore properties from untreated wood to porous BioSiC was investigated systemically. The shrinkage and weight gain during pyrolysis was discussed. It was found that the structure of as-synthesized BioSiC was related to the microstructure of wood aerogel template and the concentration of PCS precursor. The proper microstructure of cellulose skeleton which was essential for the infiltration process could obtained by removing lignin and hemicellulose appropriately. The optimum PCS content was 40 wt. % for easy infiltration and proper SiC content. The results revealed that the dredged skeleton of cellulose was reproduced perfectly by PCS ceramization. The obtained BioSiC presented high porosity (61.03%) and low density (0.86 g/cm^3^) with good Darcy permeability (19.22 mD).

## 1. Introduction

As a low cost natural renewable resource, wood have evolved hierarchical porous structures optimized by nature. Over the recent two decades wood has been commonly used for processing porous inorganic materials as biotemplate for their biological porous structures [1,2,3,4,5]. Among them, biomorphic Silicon Carbide (BioSiC) is the most attracting one. For silicon carbide (SiC) is a covalent bond compound with diamond-like structure. The physical and chemical stability of SiC is high and it can keep good mechanical strength under extreme heat [6,7]. BioSiC can inherit these properties from SiC, and the introduction of wood biotemplate can significantly simplify pore forming procedure and free of the use of bonding agents. That makes porous BioSiC a prominent candidate for applications as filtration media, catalyst support, electrode, etc. [8,9,10,11]. Despite many successes, BioSiC derived directly from nature wood template has been suffering from unsatisfied permeability and interconnectivity. This can be attributed to anisotropic of wood with tubular structure along axial direction, and extractives, pits and other wood anatomical structures blocking the channels fluid through after wood being cut down and dried. As a result, wood template and the final product have relatively high porosity but closed pores [12,13].

**Figure 1:**
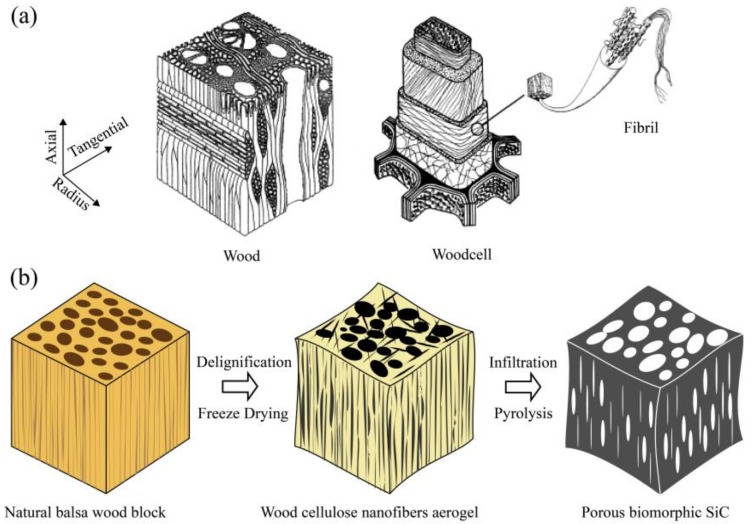
(**a**) Hierarchical microstructure of wood (reproduced with permission from Ref [1]) (**b**) illustration for the preparation of the biomorphic Silicon Carbide (BioSiC).

Wood can be regarded as composite with three main components: cellulose, hemicellulose and lignin. Cellulose constitutes the skeleton, promising the tensile strength of wood. Hemicellulose and lignin fill in the space acting as matrix material and combining nanoscale cellulose fibers together (Figure 1a). Interestingly, the wood can sustain 3D porous skeleton of cellulose nanofibers after removal of lignin and hemicellulose and thus format interconnected aerogel structures [14,15]. The wood aerogel is light in weight, highly porous and with absorbing ability [16]. It will be an effective way to achieve superior permeability and interconnectivity of BioSiC using cellulose nanofibers aerogel as biotemplate with the precursor-solution infiltration and pyrolysis (PIP) method.

In the present study, we developed a facile yet effective method to fabricate BioSiC with highly interconnected porous structure. Balsa wood (*Ochroma lagopus*) cellulose nanofibers aerogel was fabricated by removal of lignin and hemicellulose in natural wood. The cellulose aerogel was introduced as a biotemplate for fabricating BioSiC, while polycarbosilane (PCS) was chosen as the SiC ceramic precursor. Influences of aerogel template microstructure and PCS precursor content on morphology and chemical composition of the as-synthesized BioSiC were discussed. The structure and properties of cellulose aerogel templated BioSiC were investigated systematically.

## 2. Experimental 

### 2.1. Materials

The balsa wood (*Ochroma lagopus*) was purchased from Guangzhou Sinokiko Balsa Co. Ltd., Guangzhou, China. Polycarbosilane (PCS) was brought from Ningbo Zhong Xing New Materials Co. Ltd., Ningbo, China. NaClO_2_, Na_2_SO_3_, NaOH, H_2_O_2_ and xylene were purchased from Beijing Chemical Works, Beijing, China.

### 2.2. Modification of Natural Wood

The fabrication process of the BioSiC was illustrated in Figure 1b. Wood blocks with 20 mm cubic were cut from the sapwood of balsa timber and then carefully wrapped by medical gauze independently. Three different methods were conducted to treat the wood blocks in order to obtain wood cellulose aerogels with different chemical composition and microstructure. For NaOH Method, the samples were put into a beaker with mixed aqueous solution containing 2.5 mol/L NaOH and 0.4 mol/L Na_2_SO_3_ and boiled for 20 h followed by treatment with 30 wt. % H_2_O_2_ aqueous solution at 100 °C for 9 h. This method could remove most of the lignin and part of the hemicellulose in balsa wood. For NaClO_2_ Method, the wood samples were immersed directly in 10 wt. % NaClO_2_ aqueous solution for 7 days at room temperature to remove most of the lignin. The Combined Method was to treat the wood blocks with NaOH Method and NaClO_2_ Method successively, intending to confirm the superposition effect. After chemical treatments, all the balsa wood blocks were cooked in boiling deionized water for 3 h to remove residual chemicals (change water frequently). Finally, these blocks were freeze dried for 24 h to get wood cellulose nanofibers aerogels.

### 2.3. Synthesis of Biomorphic Porous SiC

The aerogels were cut into 3mm thick slices and then impregnated with 30–50 wt. % preceramic precursor polycarbosilane (PCS) xylene solvent for 12 h in vacuum atmosphere. After impregnation the solvent is removed, and the slices were placed in furnace with graphite crucible for pyrolysis: firstly, the samples were heated from room temperature to 1200 °C at a ramp rate of 20 °C/min, in the next 30 min the samples were further heated to 1500 °C. The temperature was maintained at 1500 °C for 1 h and then cooled to room temperature. Of note, the whole pyrolysis process is under the protection of Ar flux.

### 2.4. Characterizations

The morphology and chemical content of biomorphic porous SiC was studied by scanning electron microscopes (SEM/EDS, VEGA3 from TESCAN, Brno, South Moravian Region, Czech Republic and JSM-6700F, equipped with electron diffraction spectrometry, JEOL, Akishima, Tokyo, Japan). Fourier transform infrared spectroscopy (FT-IR, Spotlight 400, Perkin Elmer, Waltham, MA, USA) was used to analyze the change of functional groups of balsa wood before and after delignification. Phase composition of prepared SiC material was evaluated by X-ray Diffraction (XRD, XPert Pro MPD, PANalytical, Zürich, Switzerland) using a Cu-Kα radiation with 2θ range 10–80° at a speed of 3.5°/min. Pore structure properties for macropores were determined by mercury intrusion porosimetry method using AutoPore IV 9500 from Micromeritics (Norcross, GA, USA). Specific surface areas measurements were performed on BELSORP-max from Microtrac BEL (Osaka, Japan) and analyzed by Brunauer–Emmett–Teller method for mesopores.

## 3. Results and Discussion

### 3.1. Morphology and Chemical Analysis

**Figure 2:**
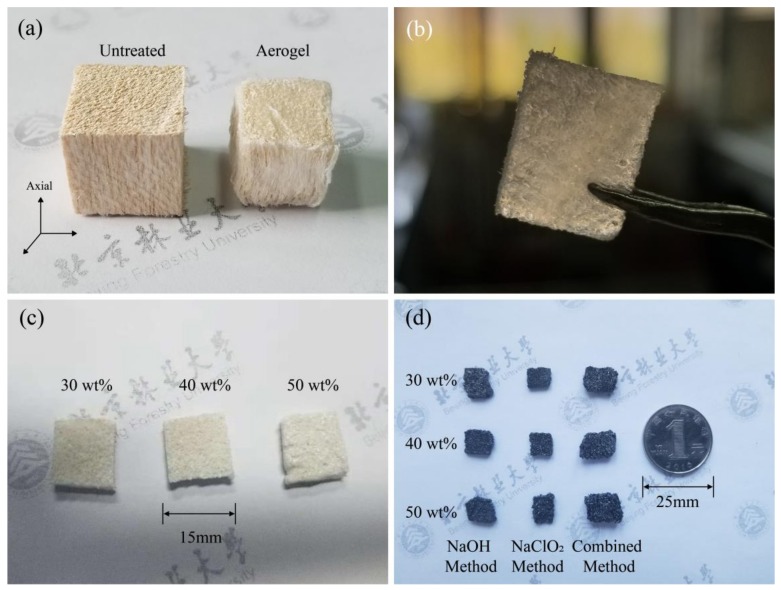
Morphology of the biotemplate (**a**), infiltrated aerogel template before and after solvent removal (**b**,**c**) and as-synthesized BioSiC (**d**).

Figure 2a demonstrates the appearance of untreated balsa wood block vs. obtained cellulose aerogel. After chemical treatment, there was a slight shrinkage in volume due to the removal of chemical components and moisture of wood. Besides, the integrity of wood texture was damaged, wood microfibers can be observed arranged in bundles thus visible voids generated between wood fibers. The chemicals promoted the dispersion of microfibers and then broken most of the wood cell wall structure. As a result, 3D interconnected cellulose nanofibers aerogel skeleton was obtained. Figure 2b shows the aerogel template infiltrated with PCS precursor solution. The template slice saturated with precursor solution was semitransparent due to the removal of brown-colored lignin, this also indicated that the solution could entirely infiltrate the template. After solvent removal by heating, PCS was recrystallized and turned from transparent to white. This could partly indicate the deposition position of PCS precursor. As illustrated in Figure 2c, the surface roughness of the template was related to the PCS solution concentration. The template surface became smoother with the increase in PCS content. That is because the viscosity of PCS solution is positive correlated with PCS content. The solution became too viscose and difficult to be removed when the PCS content reached 50 wt. %. It left a thin and smooth residual PCS layer on the surface of the template. The PCS solution has good fluidity with lower PCS content (30 wt. % and 40 wt. %). The precursor can be well impregnated into the template with the help of capillary effect and left a rough surface of the template. Figure 2d displays the as-synthesized BioSiC with different method and PCS content. The ceramic reproduced the structure of cellulose aerogel perfectly with a porous structure after pyrolysis. The volume and weight changes of BioSiC vs. aerogel template were calculated and presented in Table 1. The changes in volume and weight of BioSiC fabricated with Combined Method showed different tendency compare to those with the other two methods. Combined Method BioSiC gained less weight and volume shrinkage. It indicated less precursor infiltrated into the template. It could probably because Combined Method aerogel templates have different microstructure and difficult to be infiltrated. As to the specimens of other two methods, the volume shrinkage first increased and then decreased with the increase of weight gain. The coating layer formed on the template surface with higher PCS content may generate stress restrain the shrinkage of inside template during pyrolysis. Interestingly, BioSiC using NaOH Method gained more weight but shrank less, while BioSiC using NaClO_2_ Method behaved on the contrary. Firstly, it proved that templates using NaOH Method have better absorbing ability of precursor solution than templates using NaClO_2_ Method. Secondly, the components left in the aerogel template also affected the shrinkage of BioSiC during pyrolysis. Both of NaOH Method and NaClO_2_ Method can remove most of the lignin in wood, while NaOH Method also destroy hemicellulose. The less shrinkage of the templates treated with NaOH Method during pyrolysis could attributed to less wood chemical components left. This could also explain the different shrinkage behavior of Combined Method BioSiC, for Combined Method excessively removed lignin and hemicellulose in template.

**Figure 3:**
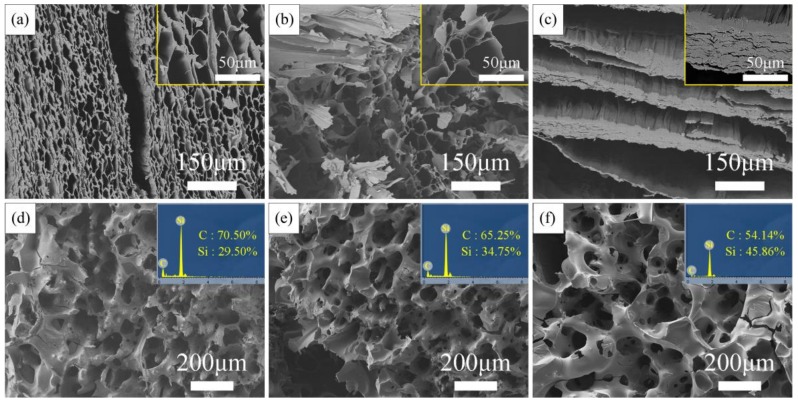
Morphology of the biotemplates from NaOH Method (**a**), NaClO_2_ Method (**b**), Combined Method (**c**) and SEM/EDS images of NaClO_2_ Method BioSiC with PCS content 30–50 wt. % (**d**–**f**).

The SEM images of the wood aerogels fabricated with different modifications are shown in Figure 3a–c. The basic porous structure of balsa wood remained in the aerogels with pretreated using the NaOH Method and the NaClO_2_ Method. The sponge-like porous structure can be impregnated with PCS solution easily. The aerogel pretreated with NaOH Method showed larger pores, and the wood cell wall can be seen damaged seriously. The aerogel pretreated with the NaClO_2_ Method showed smaller pores and more regular structure. It indicated less damage on wood cell structure. Therefore, the NaOH Method aerogel could absorb more PCS precursor. The pores in the aerogel treated with the Combined Method almost squashed when slicing due to the excess removal of lignin and hemicellulose. Therefore, the layered template is difficult to be fully infiltrated. The chemical composition differences can be further evidenced by FT-IR analysis.

The morphology and chemical composition evolution of BioSiC derived from various PCS solution concentration (30–50 wt. %) are displayed in Figure 3d–f (NaClO_2_ Method BioSiC, fracture surface). Si element was detected both on wood cell wall and cell cavity (using multiple sampling points). Considering the damage of chemical treatments on wood cell, the precursor solution can permeate into the cell cavity through the damaged cell wall, coat on the cell wall and then infiltrate it. The C:Si ratio gradually approached 1:1 with the increase in PCS content. The pore structure was irregular in shape at lower PCS content (30 wt. %). Besides, the as-synthesized BioSiC was mainly composed of carbon, which was more like pyrolyzed cellulose aerogel template rather than biotemplating ceramic. While at high PCS content (50 wt. %) the pores have bubble shape and thick wall, which structure was obviously different from wood. These pores might originated from PCS soften and foam during pyrolysis. Therefore, the present results suggested that BioSiC with proper pore structure and SiC content could obtained with 40 wt. % PCS solution.

**Figure 4:**
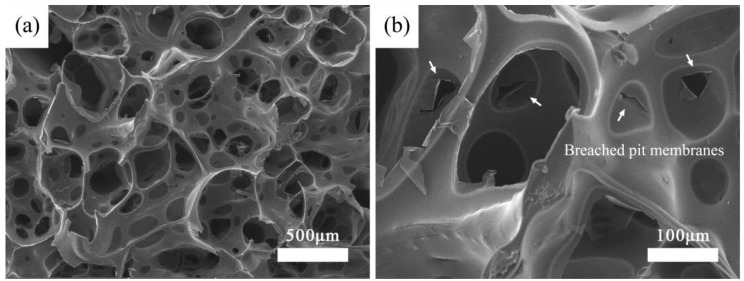
SEM images of BioSiC fabricated with NaOH Method with 40 wt. % PCS (**a**) 100× and (**b**) 400×.

Figure 4a shows the low magnification fracture surface SEM image of the BioSiC fabricated with NaOH Method with 40 wt. % PCS. As can be seen, disordered porous structure with most interconnected pores derived from balsa cellulose aerogel template was preserved. Compared with Figure 3e (NaClO_2_ Method BioSiC with same PCS content), NaOH Method BioSiC showed more open pores and better preserved the wood aerogel morphology. More importantly, most of the pores were interconnected. The produced pores can be roughly distinguished into two sizes: macropores originated from collapsed cell wall and mesopores derived from opened pits. Figure 4b exhibits the morphology of one particular pore. The penetrated pit membranes and swelled broken cell walls can be observed. The pore wall was thin and smooth. These contributed to the increase in pore openness of as-synthesized BioSiC. It was worthy of note that there were still some closed pores in the BioSiC. From their round-shaped edge and concaved membrane-like structure in central, these closed sections can be identified as unbreached pit membranes. The main chemical composition of pit membranes was cellulose, for technically pit membranes were part of primary wall of wood cell. In other words, the lignin and hemicellulose content in these sections was relatively low compared with secondary wall. The chemical treatments adopted to remove lignin and hemicellulose have weaker influence on pit membranes and that could explain the closed pores in BioSiC. From the results presented above, NaOH Method aerogel template with 40 wt. % PCS is the optimum way to fabricate BioSiC with high porous and interconnected pore structure and proper SiC content.

### 3.2. FT-IR Spectroscopy Analysis

**Figure 5:**
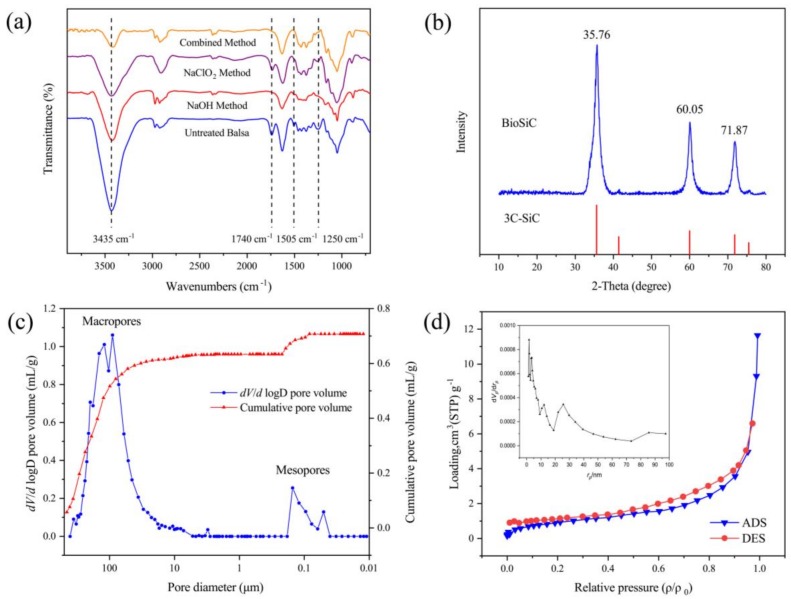
FT-IR spectra (**a**), XRD pattern (**b**), MIP pore size distribution (**c**), and BET&BJH plot (**d**).

The difference in chemical compositions of wood cells on NaOH Method and NaClO_2_ Method treatment was confirmed through FT-IR analysis in Figure 5a. The absorption peak at 1505 cm^−1^ which represents the benzene skeleton vibration of lignin disappeared in all chemical treated specimens. It indicated the removal of lignin [17]. The absorption peaks at 1740 cm^−1^ assigned to C=O stretching of hemicellulose disappeared after treated with NaOH Method and Combined Method. It indicated the removal of hemicellulose. The absorption peak at 1250 cm^−1^ represents CO–OR stretching of hemicellulose and C–O stretching vibration of phenolic hydroxyl from lignin. It decreased in NaClO_2_ Method specimen but disappeared in the other two methods’ specimens. This can further evidence that the NaOH Method can remove both of lignin and hemicellulose, while the NaClO_2_ Method only affects the lignin. The peak at 3435 cm^−1^ is relevant to carboxyl groups in cellulose, hemicellulose, and lignin. The decrease in the intensity of the peak when treated with Combined Method demonstrated the removal of hemicellulose and lignin. Additionally, the crystallinity (K) of cellulose chains in NaOH Method aerogel could be roughly calculated according to well recognized Nelson’s method (K = A_1372_/A_2900_) [18], where K is the infrared ratio of two characteristic peaks: 1372 cm^−1^ for C–H bending and 2900 cm^−1^ for C–H stretching. The calculated crystallinity decreased from 1.7852 of untreated balsa wood to 1.6802 after NaOH Method treatment. It can be inferred that some microfibers separated from each other and arranged in disordered status without lignin and hemicellulose.

### 3.3. XRD Analysis

Figure 5b shows the crystalline phase composition of the prepared biomorphic ceramic. There are three main diffraction peaks with 2θ values of 35.76°, 60.05°and 71.87°, corresponding to the 111, 220 and 311 crystal planes of 3C β-SiC, comparing with the standard PDF card of SiC (JSPDS #29-1129) [19]. The crystallinity (63.24%) and grain size (110 nm) were also obtained by software. These results are consistent with previous studies that biotemplate can restrain grain growth, which is propitious for the material to sustain mechanical strength with high porosity [20].

### 3.4. Pore Properties

Mercury intrusion porosimetry (MIP) was carried out for further determination of the properties of pore structures in NaOH Method BioSiC with 40 wt. % PCS and the main characteristics were summarized in Table 2. As shown in Figure 5c, there is a bimodal pore size distribution in this produced porous material. The pores can be classified as macropores with average diameter around 100 μm and mesopores around 0.1 μm. The macropores dominated, which take as high as 90%. The pore size distribution is similar to previous study of untreated balsa wood [21]. It illustrated that the natural tubular pore structure fabricated by vessels and wood fibers was partly destroyed along with the disintegration of wood cell wall after delignification. Aerogel sponge-like structure with axial and transverse pores was formed from cellulose nanofibers. These fabricated pores can allow fluids permeating. The structure was reproduced perfectly by PCS infiltration and pyrolysis process. The Darcy air permeability of the produced BioSiC was 19.22 mD, which was calculated via Katz and Thompson model based on MIP data [22,23]. The permeability is as good as high quality sandstone, which can reflect the improvement of interconnectivity of novel BioSiC. The BET (Brunauer–Emmett–Teller) specific surface area and BJH (Barrett–Joyner–Halenda) pore size distribution were measured using N_2_ adsorption (Figure 5d). The plot shows a typical type IV adsorption isotherm and the specific surface area is 3.5175 m^2^ g^−1^. This is probably due to the shrinkage volume during pyrolysis, which caused the closure of micropores between microfibers. The skeletal density was measured 2.2139 g/cm^3^, which was lower than that of pure SiC (3.2 g/cm^3^). It indicated there is still a small amount of amorphous carbon in the ceramic, which could be further activated to increase the volume of micropores and act as active position.

## 4. Conclusions

The porous biomorphic SiC ceramic with high interconnectivity and permeability was fabricated from cellulose nanofibers aerogel of balsa wood. It was essential to remove both lignin and hemicellulose appropriately to obtain wood aerogel with interconnected pores. The structure of as-synthesized BioSiC was related to the microstructure of wood aerogel template and the concentration of PCS precursor. The microstructure of aerogel template was found correlated with absorbing ability of PCS precursor solution, which could partly affect the difficulty of infiltration and shrinkage during pyrolysis. The infiltration difficulty and shrinkage were also found to be related with PCS concentrations in the precursor solution. More PCS content contributed to higher Si to C ratio in BioSiC, but lower solution fluidity to fully infiltrate the template and affected the replicate of biotemplate. The optimum PCS content was 40 wt. % for easy infiltration and proper SiC content. The synthesized BioSiC hold the pores structure axial of natural wood and transverse pores of aerogel structure. The remained closed pores in BioSiC were originated from closed pit membranes that were slightly influenced by delignification treatment. The synthesized BioSiC exhibited large pore size, high porosity (61.03%) and good Darcy permeability (19.22 mD), which was potential for filtration and thermal insulation.

## Figures and Tables

**Table 1:** The volume and weight changes of the BioSiC compared with templates before infiltration.

Treatment Methods	Volume Shrinkage			Weight Gain		
	30 wt. %	40 wt. %	50 wt. %	30 wt. %	40 wt. %	50 wt. %
NaOH Method	65.72%	66.24%	60.36%	134.72%	148.36%	152.70%
NaClO_2_ Method	78.85%	82.84%	78.42%	110.16%	122.39%	132.91%
Combined Method	58.87%	52.27%	47.43%	38.63%	63.08%	53.60%

**Table 2:** The pore parameters of the BioSiC determined by MIP.

Porosity (%)	Median Pore Diameter (Volume) (μm)	Median Pore Diameter (Area) (nm)	Bulk Density (g/cm^3^)	Skeletal Density (g/cm^3^)
61.0252	99.0509	100.1	0.8629	2.2139

## References

[1] Greil P., Lifka T., Kaindl A. (1998). Biomorphic Cellular Silicon Carbide Ceramics from Wood: I. Processing and Microstructure. J. Eur. Ceram. Soc..

[2] Sun B., Fan T., Zhang D. (2002). Porous TiC ceramics derived from wood template. J. Porous Mater..

[3] Cao J., Rambo C.R., Sieber H. (2004). Preparation of Porous Al_2_O_3_-Ceramics by Biotemplating of Wood. J. Porous Mater..

[4] Ramírez-Rico J., Martínez-Fernandez J., Singh M. (2017). Biomorphic ceramics from wood-derived precursors. Int. Mater. Rev..

[5] Pullar R.C., Novais R.M. (2017). Cork-based biomimetic ceramic 3-DOM foams. Mater. Today.

[6] Han F., Zhong Z., Yang Y., Wei W., Zhang F., Xing W., Fan Y. (2016). High gas permeability of SiC porous ceramics reinforced by mullite fibers. J. Eur. Ceram. Soc..

[7] Wei W., Zhang W., Jiang Q., Xu P., Zhong Z., Zhang F., Xing W. (2017). Preparation of non-oxide SiC membrane for gas purification by spray coating. J. Membr. Sci..

[8] Gómez-Martín A., Orihuela M., Becerra J., Martínez-Fernández J., Ramírez-Rico J. (2016). Permeability and mechanical integrity of porous biomorphic SiC ceramics for application as hot-gas filters. Mater. Des..

[9] Wang H., Schmack R., Paul B., Albrecht M., Sokolov S., Rümmler S., Kondratenko E.V., Kraehnert R. (2017). Porous silicon carbide as a support for Mn/Na/W/SiC catalyst in the oxidative coupling of methane. Appl. Catal. A.

[10] Li W., Liu Q., Chen S., Fang Z., Liang X., Wei G., Wang L., Yang W., Ji Y., Mai L. (2018). Single-crystalline integrated 4H-SiC nanochannel array electrode: Toward high-performance capacitive energy storage for robust wide-temperature operation. Mater. Horiz..

[11] Istomina E.I., Istomin P.V., Nadutkin A.V. (2013). Preparation of biomorphic SiC. Inorg. Mater..

[12] Diaz-Rodriguez P., Landin M., Rey-Rico A., Couceiro J., Coenye T., Gonzalez P., Serra J., López-Álvarez M., León B. (2011). Bio-inspired porous SiC ceramics loaded with vancomycin for preventing MRSA infections. J. Mater. Sci. Mater. Med..

[13] Lee D.J., Jang J.J., Park H.S., Kim Y.C., Lim K.H., Park S.B., Hong S.H. (2012). Fabrication of biomorphic SiC composites using wood preforms with different structures. Ceram. Int..

[14] Song J., Chen C., Yang Z., Kuang Y., Li T., Li Y., Huang H., Kierzewski I., Liu B., He S. (2018). Highly Compressible, Anisotropic Aerogel with Aligned Cellulose Nanofibers. ACS Nano.

[15] Song J., Chen C., Zhu S., Zhu M., Dai J., Ray U., Li Y., Kuang Y., Li Y., Quispe N. (2018). Processing bulk natural wood into a high-performance structural material. Nature.

[16] Guan H., Cheng Z., Wang X. (2018). Highly Compressible Wood Sponges with a Spring-like Lamellar Structure as Effective and Reusable Oil Absorbents. ACS Nano.

[17] Huang Y., Chen Y., Fan X., Luo N., Zhou S., Chen S.-C., Zhao N., Wong C.P. (2018). Wood Derived Composites for High Sensitivity and Wide Linear-Range Pressure Sensing. Small.

[18] Nelson M.L., O’Connor R.T. (1964). Relation of certain infrared bands to cellulose crystallinity and crystal lattice type. Part II. A new infrared ratio for estimation of crystallinity in celluloses I and II. J. Appl. Polym. Sci..

[19] Gou Y., Wang H., Jian K., Shao C., Wang X. (2017). Preparation and characterization of SiC fibers with diverse electrical resistivity through pyrolysis under reactive atmospheres. J. Eur. Ceram. Soc..

[20] Zhang W., Zhang B., Jin H., Li P., Zhang Y., Ma S., Zhang J. (2018). Waste eggshell as bio-template to synthesize high capacity δ-MnO_2_ nanoplatelets anode for lithium ion battery. Ceram. Int..

[21] Gross K.A., Rodriguez-Lorenzo L.M. (2012). Assessment of the micropore system in wood for drug delivery. J. Aust. Ceram. Soc..

[22] Katz A.J., Thompson A.H. (1986). Quantitative prediction of permeability in porous rock. Phys. Rev. B.

[23] Gao Z., Hu Q. (2013). Estimating permeability using median pore-throat radius obtained from mercury intrusion porosimetry. J. Geophys. Eng..

